# Expanding Horizons in Syphilis Treatment: Challenges, Advances, and Opportunities for Alternative Antibiotics

**DOI:** 10.1007/s11904-025-00725-4

**Published:** 2025-03-13

**Authors:** Diana D. Villarreal, Chibuzor M. Babalola

**Affiliations:** 1 Department of Pediatrics, Maternal Child and Adolescent/Adult Center for Infectious Disease and Virology, University of Southern California, 1100 N. State Street, IRD112, Los Angeles, CA 90033 USA; 2 Department of Population and Public Health Sciences, Keck School of Medicine, University of Southern California, Los Angeles, CA USA

**Keywords:** Syphilis, Syphilis Therapy, Alternative Antibiotics

## Abstract

**Purpose of Review:**

This review examines the growing need for alternative technologies to address the resurgence of syphilis, particularly its congenital and late-stage manifestations. It explores current treatment paradigms, highlights the limitations of penicillin, and evaluates emerging evidence on new therapies and diagnostics to inform future strategies.

**Recent Findings:**

Recent breakthroughs in *Treponema pallidum* culture techniques have enabled antibiotic susceptibility testing, expanding knowledge on both established and emerging treatment options. Alternatives like ceftriaxone, doxycycline, cefixime, and dalbavancin show promise, with other candidates in trials, though evidence is limited beyond early-stage syphilis. Shortened penicillin regimens also challenge historical assumptions about treatment duration. Advanced molecular diagnostics may complement currently limited serologic monitoring to improve evaluations in healthcare and research.

**Summary:**

While penicillin remains effective, its limitations necessitate alternatives. Emerging antibiotics and improved diagnostics offer opportunities to simplify treatment and enhance care. Future robust trials should validate new treatments, refine dosing strategies, and integrate innovative diagnostics, particularly including underserved and vulnerable populations.

**Supplementary Information:**

The online version contains supplementary material available at 10.1007/s11904-025-00725-4.

## Introduction

Syphilis, caused by *Treponema pallidum (T. pallidum)*, continues to be a major global public health concern. Despite being preventable and treatable, the disease has seen a troubling resurgence in recent decades [1]. Globally, the number of incident syphilis cases increased from approximately 8.8 million in 1990 to over 14 million in 2019, with the age-standardized incidence rate rising from 160 to 178 per 100,000 persons during this period [2]. In the United States, syphilis cases nearly doubled between 2018 and 2022, reaching the highest number of reported infections in 70 years [3]. This resurgence is evident across various regions globally, including Europe, Canada, and Australia, affecting diverse populations.

Congenital syphilis, transmitted from mother to child during pregnancy, is particularly devastating, often resulting in stillbirth, prematurity, or disseminated disease of the newborn, with profound long-term consequences for individuals and communities [4]. Untreated maternal syphilis leads to adverse birth outcomes in 15 − 80% of cases, depending on the stage of infection [4]. Additionally, syphilis can progress to severe complications in adults such as cardiovascular syphilis, neurosyphilis, and gummatous lesions, leading to significant long-term morbidity. A central challenge in addressing syphilis lies in its often asymptomatic or latent presentation in infected adults and infants, which hinders timely diagnosis and complicates treatment monitoring [5].

The rise and resurgence of syphilis, beyond socio-demographic factors or social determinants, indicates gaps in clinical detection, treatment, and management. Multiple factors have contributed to the resurgence, including a global penicillin shortage, reliance on historical treatment paradigms, and the lack of potent and reliable alternative therapies [6–8]. Addressing these challenges requires a re-evaluation of treatment and diagnostic paradigms.

The increase in syphilis cases underscores the need to recognize the disease's stages, especially in populations with complex presentations, and rethink treatment algorithms to accommodate diverse patient needs. Syphilis progresses through distinct clinical stages if left untreated (Table 1), and the complexity of diagnosing, treating, and monitoring these stages, especially in key populations can exacerbate the challenge [9]. Syphilis typically begins with primary syphilis, presenting as a painless chancre, which can progress to the systemic symptoms and mucocutaneous lesions characteristic of secondary syphilis. If untreated, the infection may enter a latent phase, remaining asymptomatic or progressing to tertiary syphilis, characterized by granulomatous inflammation affecting various organ systems, including the cardiovascular and central nervous systems. Neurosyphilis, which can occur at any stage, further complicates management, and presents in diverse ways such as meningitis, stroke, or dementia [9]. In congenital syphilis, missed or delayed diagnoses can result in devastating complications to individuals and the society at large [4]. Recognizing the stages of infection or population-specific disease presentations is crucial, but alone it does not address the broader challenges of syphilis management. Importantly, the entire cascade of syphilis management is shaped by the reliance on a single gold-standard antibiotic — penicillin.

**Table 1:** Stages of Syphilis

Stage	Timing After Exposure	Symptoms
Primary Syphilis	3–6 weeks	Painless chancre at the site of infection, regional lymphadenopathy
Secondary Syphilis	6 weeks to 6 months	Generalized rash (often including palms and soles), mucous patches, condylomata lata, fever, malaise, lymphadenopathy
Early Latent Syphilis	< 1 year	Asymptomatic but serologically positive
Late Latent Syphilis	> 1 year	Asymptomatic but serologically positive
Early Neurosyphilis	Weeks to months	Headache, meningitis, cranial nerve dysfunction, auditory or ocular symptoms, stroke-like symptoms
Late Neurosyphilis	Years to decades	Tabes dorsalis, general paresis, Argyll Robertson pupil (accommodates but does not react to light)
Tertiary Syphilis	1–30 years	Gummas (granulomatous lesions), cardiovascular syphilis (aortitis, aneurysms), neurosyphilis signs if untreated earlier
Early Congenital Syphilis	Birth to 2 years	Hepatosplenomegaly, jaundice, nasal discharge ('snuffles'), rash, pseudoparalysis, bone changes (osteochondritis)
Late Congenital Syphilis	> 2 years	Hutchinson's triad (interstitial keratitis, notched teeth, deafness), saddle nose, frontal bossing, Clutton's joints

Penicillin, a highly effective antibiotic, has sustained a long-standing role as the cornerstone of syphilis management across all stages. However, reliance on this single therapy amidst the lack of potent and reliable alternative treatments, limits current clinical management approaches. The efficacy of penicillin against syphilis is not in question; however, its drawbacks — including the need for injectable administration, lengthy treatment courses, and potential barriers such as allergies or supply chain issues—pose significant challenges to patient adherence and equitable access, especially where resources are limited. These barriers were compounded by the historical inability to clinically isolate *T. pallidum* and test for antibiotic susceptibility, which has forced treatment decisions to rely heavily on historically successful penicillin-based treatment paradigms rather than tailored clinical decisions. The constraint of such reliance becomes most apparent particularly in situations where penicillin is unavailable from supply chain or logistical barriers, when simplified regimens are needed to address co-infections, or when alternative modes of administration, such as oral therapies, could improve patient adherence. There is a critical need for robust, evidence-based alternative therapies that can match penicillin's efficacy while offering greater versatility and accessibility.

Fortunately, recent advancements in *T. pallidum* culture techniques and antibiotic susceptibility testing offer opportunities to expand treatment options and refine approaches. This manuscript discusses the current treatment standards for syphilis, highlights the evidence base for specific treatment regimens, and explores alternative antibiotics and treatment durations. We also emphasize the need for further work to address existing gaps in evidence and propose strategies for advancing syphilis treatment options, particularly in special populations.

## Monitoring Treatment Success

In addressing these treatment gaps, the ability to accurately monitor treatment success becomes essential, particularly when evaluating alternative therapies for syphilis. While the need for alternative antibiotics is clear, where diagnostic or monitoring tools are inadequate, even effective new therapies may not demonstrate their full value.

Currently, monitoring treatment efficacy in syphilis clinically relies on serologic tests, primarily nontreponemal tests such as the rapid plasma reagin (RPR) and Venereal Disease Research Laboratory (VDRL) tests. These tests measure antibody titers directed against cardiolipin, a lipid released during cellular damage and inflammation, providing an indirect measure of disease activity and response to treatment [10]. In contrast, treponemal tests detect antibodies directed specifically against *T. pallidum* antigens, which typically remain positive for life, making them less useful for monitoring treatment response or disease activity, since a person can be cured or reinfected and they will remain positive [10]. The standard criterion for successful treatment is a fourfold decline in nontreponemal titers by 6 to 12 months, with a nonreactive nontreponemal test considered the best evidence of cure [10]. However, nontreponemal serologies are often unreliable due to false elevations and variability in titers and post-treatment decay. Many patients fail to achieve a nonreactive status despite effective therapy, complicating the assessment of both current and alternative regimens [11]. Animal studies and clinical observation have shown RPR decay without treatment, complicating the utility of this test [12–14].

The challenge of monitoring syphilis treatment is compounded further by the often asymptomatic or latent nature of the disease. Unlike many bacterial infections, the treatment success of syphilis cannot be monitored through symptom resolution, leaving serologic tests as the primary indicator. However, these tests are not always sufficient to evaluate outcomes, particularly in populations with advanced or congenital syphilis. For instance, asymptomatic pregnant individuals with latent syphilis may unknowingly transmit the infection vertically, and infants with undetected congenital syphilis may develop irreversible sequelae of late congenital syphilis [4]. Approximately 15% of adults with latent syphilis can progress to tertiary syphilis years or decades after the initial infection [15]. These limitations underscore the need for improved diagnostic tools, including direct detection methods, to more accurately evaluate treatment outcomes and guide clinical decisions with both existing and emerging treatments.

Emerging molecular diagnostic tools, such as polymerase chain reaction (PCR)-based assays or novel antigen-detection assays, offer promising avenues to address these challenges [16]. By enabling direct detection of *T. pallidum*, these methods could complement serologic testing, providing greater precision in monitoring treatment success. Such tools are particularly relevant for effective evaluations of alternative antibiotics where precise standardized measures of response are essential such as with advanced and congenital syphilis [13]. Incorporating these advanced diagnostic tools future studies of alternative therapies would improve the reliability of treatment outcome assessments and guide their integration into clinical care.

## Penicillin in Syphilis Treatment: Current Recommendations and Limitations

### Treatment Algorithms Across Syphilis Stages

Both the World Health Organization (WHO) and the U.S. Centers for Disease Control (CDC) provide treatment recommendations for syphilis that vary by stage and whether central nervous system involvement is present (Table 2) [9, 17, 18]. Early syphilis is typically treated with a single intramuscular dose of benzathine penicillin G. Late latent and tertiary syphilis require three weekly doses of benzathine penicillin G, while neurosyphilis and ocular syphilis necessitate intravenous penicillin or ceftriaxone for 10 to 14 days [9]. Despite subtle changes in formulation and dosage over the past 70 + years, penicillin has long been established as the gold standard for syphilis management [9].

**Table 2:** Current Recommended Treatments for Syphilis

Stage/Category	Recommended Treatment
Primary & Secondary Syphilis	Benzathine Penicillin G 2.4 million units IM once; Doxycycline 100 mg orally BID for 14 days if penicillin-allergic
Early Latent Syphilis	Benzathine Penicillin G 2.4 million units IM once; Doxycycline 100 mg orally BID for 14 days if penicillin-allergic
Late Latent Syphilis	Benzathine Penicillin G 2.4 million units IM weekly for 3 weeks; Doxycycline 100 mg orally BID for 28 days if penicillin-allergic
Tertiary Syphilis	Benzathine Penicillin G 2.4 million units IM weekly for 3 weeks; Ceftriaxone 1–2 g IV/IM daily for 10–14 days for penicillin-allergic
Neurosyphilis	Aqueous Penicillin G 18–24 million units/day IV for 10–14 days; Ceftriaxone 2 g IV daily for 10–14 days for penicillin-allergic
Ocular Syphilis	Same as Neurosyphilis treatment
Congenital Syphilis	Aqueous Penicillin G 100,000–150,000 units/kg/day IV divided every 8–12 h for neonates, for 10 days
Pregnant Women	Same as corresponding syphilis stage; Benzathine Penicillin G is the only recommended antibiotic due to safety in pregnancy; Doxycycline is not recommended during pregnancy

### Effectiveness and Limitations of Penicillin

*T. pallidum* continues to demonstrate high susceptibility to first-generation penicillin, as demonstrated by both clinical and in vitro studies. Depot preparations of benzathine penicillin G achieve prolonged, stable levels of penicillin, effectively eradicating the organism despite its slow replication [19–26]. However, the reliance on penicillin introduces critical vulnerabilities.

Approximately 10–20% of patients do not achieve the standard serological marker of treatment success—a fourfold titer reduction in nontreponemal tests—by 12 months [10, 26]. These incomplete responses indicate the challenges in relying solely on serological outcomes to assess treatment efficacy. Furthermore, penicillin-based regimens face adherence challenges, particularly in resource-limited settings where multi-dose schedules for late latent syphilis may be difficult to complete. Intramuscular injections, while effective, can deter patients due to pain or logistical barriers [27].

### Gaps in Evidence for Special Populations

The most robust evidence supports penicillin use in early syphilis, but data for late-stage syphilis, neurosyphilis, and congenital syphilis remain sparse [17, 28]. For individuals living with HIV, co-infection may impair treatment efficacy, necessitating closer follow-up. However, current evidence does not indicate the need for differing treatment guidelines [9, 26]. The paucity of data limits evidence-based decision-making and underscores the need for further research to optimize treatment strategies for these populations.

### Challenges in Implementation and Access

For patients allergic to penicillin, oral doxycycline is available. However, many socially vulnerable patients have difficulty completing a 14 or 28-day course of twice daily doxycycline. Penicillin desensitization protocols are an option but are resource-intensive and often impractical in many healthcare settings. Moreover, while depot formulations of benzathine penicillin G are effective for most syphilis stages, they fail to achieve adequate CNS levels, making them unsuitable for neurosyphilis and congenital syphilis management. Conditions requiring intravenous therapy, such as neurosyphilis or congenital syphilis, are further complicated by penicillin’s short half-life, necessitating frequent dosing. Missed doses can disrupt treatment and require restarting the entire course, increasing burdens on both patients and healthcare systems [9]. These challenges, compounded by global penicillin shortages, complicate care. There is a critical need for scalable alternatives that address access and adherence barriers [8].

### Impact on Vulnerable Populations

The lack of viable alternatives to penicillin disproportionately affects vulnerable populations. Pregnant women rely on penicillin as the only recommended treatment to prevent maternal–fetal transmission, and neonates diagnosed with congenital syphilis require prolonged intravenous therapy, which is invasive and logistically demanding. Individuals with advanced immunosuppression due to HIV may experience variations in treatment response [26]. The unique needs of these populations make the development of new accessible and effective therapies an urgent public health priority [9].

### Call for Alternatives

The above gaps underscore the urgent need for alternatives to complement or replace penicillin in specific scenarios. Such alternatives could address barriers related to adherence, co-infections, and logistical challenges, while improving outcomes for underserved and vulnerable populations.

## Alternative Antibiotics: Clinical and In Vitro Evidence

### Established Alternatives and Their Clinical Role

Established alternatives to penicillin, including ceftriaxone, doxycycline, and amoxicillin (often combined with probenecid), have demonstrated clinical efficacy in treating syphilis, though evidence is strongest for early-stage disease [28, 29].

#### Ceftriaxone

Ceftriaxone, supported by its low minimum inhibitory concentrations (MICs), robust pharmacokinetics, and favorable clinical outcomes, has emerged as a leading option [30]. Recent systematic reviews and meta-analyses, consistently show ceftriaxone’s efficacy in achieving serologic cure rates comparable to penicillin, even in patients with HIV (Supplemental Table 1) [28, 29]. Randomized clinical trials (RCTs) reporting favorable clinical outcomes further validate ceftriaxone’s effectiveness, both for early-stage syphilis and neurosyphilis [30–42]. These findings solidify ceftriaxone as a reliable substitute in scenarios of penicillin unavailability or allergy.

#### Doxycycline

Doxycycline, an oral tetracycline, has been a practical option for penicillin-allergic patients, particularly for early-stage syphilis. Systematic reviews highlight its comparable serologic response rates to benzathine penicillin G for early syphilis, although evidence for its efficacy for neurosyphilis is limited [28–30, 43–45]. A retrospective cohort of 16 neurosyphilis patients treated with doxycycline demonstrated promising results regarding its potential efficacy in neurosyphilis treatment, though the limited sample size and lack of comparative data preclude robust conclusions [43]. Importantly, observational data and prospective studies provide much of the current evidence base for doxycycline, consistently supporting its efficacy in early-stage disease [34, 46–55]. However, its use during pregnancy has been historically constrained by concerns over teratogenicity and permanent teeth staining in the fetus. These long-standing concerns have been increasingly challenged. Recent studies highlight that the data supporting these adverse effects are outdated, not substantive, and fail to justify the exclusion of doxycycline during pregnancy, but given its historical avoidance during pregnancy, there is no evidence for its efficacy in prevention of congenital syphilis [56]. Targeted research can definitively assess its safety profile and potential role in maternal syphilis management, particularly given the lack of viable oral alternatives for this population.

#### Amoxicillin (with Probenecid)

Amoxicillin, often combined with probenecid to enhance plasma levels, has shown promise in retrospective studies, particularly in patients living with HIV [26, 57–63]. However, low-dose amoxicillin monotherapy has been ineffective in achieving serologic cure, underscoring the need for higher doses or combination therapies [57, 64]. While systematic reviews support its potential, the lack of RCTs leaves significant gaps in evidence for broader clinical use.

#### Azithromycin

Azithromycin was once considered a viable alternative for syphilis treatment, with early RCTs showing potential. However, systematic reviews and subsequent trials revealed widespread macrolide resistance and high treatment failure rates, significantly limiting its utility [65–69]. As a result, azithromycin is no longer recommended for syphilis treatment in most regions.

Despite their increasing importance, the established alternatives above have limitations [70]. There is insufficient clinical trial data to provide definitive recommendations for specific populations, such as pregnant individuals, neonates, and persons with late-stage or neuro syphilis, which has translated to continued reliance on penicillin [28, 30, 71–73]. These limitations underscore the need for additional evidence and advanced tools to optimize treatment strategies, which recent advancements in *T. pallidum* research have begun to address.

### Advancements in In vitro Testing

Recent breakthroughs in continuous *T. pallidum* culture systems have significantly advanced the study of antibiotic susceptibility, addressing long-standing challenges associated with isolating and growing the organism in vitro [74–76]. *T. pallidum* is a fragile organism that cannot survive outside a host for prolonged periods, which historically necessitated reliance on other models such as rabbit intratesticular or intradermal models for in vivo propagation and research. Until 2018, *T. pallidum* was unable to grow long-term in culture, and even now, isolation and cultivation are limited to specialized research settings, making individualized susceptibility testing unavailable in clinical microbiology laboratories [76]. As a result, susceptibility testing cannot be performed as part of routine clinical care, and treatment decisions rely heavily on the historical use of penicillin. Overall, this has limited the ability to systematically evaluate new or repurposed treatments. Now, continuous culture methods have enabled the study of susceptibility patterns across a wider range of antibiotics, including both established and emerging options.

In vitro studies by Hayes et al. and Tantalo et al. in 2023 marked a turning point by providing much-needed generalized susceptibility patterns for *T. pallidum* that can guide modern treatment strategies [74, 75]. Hayes et al. demonstrated the efficacy of beta-lactams such as piperacillin, nafcillin, and ceftriaxone, with ceftriaxone emerging as a leading alternative due to its high activity at low concentrations [75]. Tantalo et al. further confirmed ceftriaxone's exceptional potency (MIC as low as 0.0025 µg/mL), while also highlighting other fairly established options like amoxicillin and doxycycline. Lastly their study shed light on dalbavancin, a long-acting lipoglycopeptide, as a potential single-dose regimen to address adherence challenges [74]. Together, these studies represent a significant step forward in expanding and refining treatment options for syphilis, particularly in complex cases. In comparison to doxycycline which is highly accessible and already commonly employed in clinical practice, especially for penicillin-allergic patients, it is worth noting that many of the other alternatives highlighted by Hayes and Tantalo et al. are, like penicillin, parenteral and not yet widely available.

### Emerging Antibiotics

Building on the foundations laid by in vitro advancements, several antibiotics, with promising though less established clinical evidence for efficacy against syphilis infection, have emerged as potential alternatives to penicillin. These include cefixime, linezolid, and dalbavancin [77].

#### Cefixime

Cefixime, an oral cephalosporin, has demonstrated favorable pharmacokinetics and efficacy in early clinical trials. Its potential as an oral alternative for early-stage syphilis is particularly compelling. A pilot study compared patients treated with cefixime to patients treated with penicillin. In the per protocol analysis, the treatment response at 3 or 6 months was achieved by 93% (95% CI, 81%–100%; 14/15) of participants in the penicillin treatment arm and 87% (95% CI, 69%–100%; 13/15) in the cefixime treatment arm [78]. Two large randomized controlled trials are underway, aiming to validate the non-inferior safety and effectiveness of a 10-day regimen of the antibiotic compared to benzathine penicillin G [78–80]. As of December 2024, one of these cefixime studies enrolled 156 subjects with early latent syphilis (78 received cefixime, 70 received penicillin, and 8 received doxycycline). Treatment success across treatment arms by 6-months was: 89.5% for cefixime, 96.0% for penicillin, and 100% for doxycycline. Cefixime and penicillin showed similar efficacy, and the study continued [81, 82].

#### Linezolid

Linezolid, though promising in preclinical studies, has yielded mixed results in clinical evaluations. The Trep-AB study found a 5-day linezolid regimen inferior to benzathine penicillin G for early syphilis, highlighting the need for further exploration of extended regimens. In the Trep-AB study, 59 patients were randomly assigned to linezolid (n = 29) or benzathine penicillin G (n = 30). After 48 weeks follow-up, 19 of the 27 participants (70%) in the linezolid group had responded to treatment, and 28 of the 28 participants (100%) in the benzathine penicillin G group had responded to treatment. The treatment difference was calculated to be −29.6, with a 95% confidence interval of −50.5 to −8.8 [83, 84]. Ongoing studies are investigating whether longer regimens could address this limitation [77, 85]. As of October 2024, a second linezolid pilot study looking at a 10-day duration of therapy had enrolled 8 patients with early syphilis. Five patients responded to therapy, two patients were pending the 3 or 6-month timepoints, and one patient failed therapy with an increased rapid plasma regain titer at 1 month [86]. Statistical analysis was not yet available.

#### Dalbavancin

Dalbavancin, a lipoglycopeptide, stands out among emerging candidates due to its long-acting profile; a unique advantage in that it offers a single-dose regimen, which could significantly improve adherence when multi-dose regimens are challenging. In vitro studies confirm its efficacy against *T. pallidum* and suggest ability to maintain therapeutic levels for up to six weeks. The low MIC values (0·125 mg/L) compared to the concentration achieved in human plasma (19.5 mg/L at 168 h after administration) and extended half-life of dalbavancin (145 h) suggest that a single infusion could sustain high plasma concentrations long enough to achieve syphilis cure [74]. This makes dalbavancin a compelling candidate for further investigation in clinical settings, especially for vulnerable populations.

These emerging antibiotics represent promising candidates for syphilis management; dalbavancin’s long-acting profile and cefixime’s oral formulation standing out in their potential to provide choice or customizability whilst addressing adherence challenges.

#### Strengths and Gaps in the Available Evidence

Despite these advancements, significant gaps persist. The lack of robust data for certain populations, such as neonates and individuals with neurosyphilis, limits the widespread adoption of alternative regimens. Additionally, while emerging antibiotics offer exciting possibilities, comprehensive research is crucial to determine their optimal use in clinical care and inform recommendations. Dalbavancin’s long-acting profile and cefixime’s oral formulation address unique adherence challenges, but relevant evidence on safety, cost, and accessibility will be a key consideration for their widespread implementation. Addressing these gaps will require rigorous clinical trials, as well as efforts to integrate in vitro findings into practical, scalable solutions for syphilis treatment.

## Revisiting Treatment Durations and Algorithms

Alongside the crucial need for expanding the antibiotic repertoire for syphilis, optimizing treatment durations has also become an important focus of recent research. Multiple randomized clinical trials in the United States demonstrated comparable efficacy between one and three injections of benzathine penicillin G comparable for early syphilis in patients with and without HIV [87, 88]. Retrospective data also suggest no significant differences in outcomes between one and three injections of benzathine penicillin G for late latent syphilis [89]. These findings, while promising, are notably US-centric with reduced variability due to patient populations that predominantly include individuals with HIV who are well sustained on antiretroviral therapy. Nonetheless, they challenge the long-standing assumption and current practice that three weekly doses are required for late syphilis or syphilis of unknown duration, a recommendation originally based on a 1976 expert opinion [90]. Simplifying current penicillin regimens could significantly improve adherence and accessibility; however, robust evidence from additional randomized controlled trials is needed, particularly for later stages of syphilis and underrepresented populations such as pregnant women.

The question of treatment duration becomes even more critical as new therapies are explored, including existing antibiotics that could be repurposed. For instance, a 5-day course of linezolid was found to be inferior to a single injection of benzathine penicillin G for early syphilis. Rather than deterring further investigation, these findings have prompted ongoing trials evaluating 10-day regimens of linezolid, which may provide valuable new insights [77, 83]. Persisting in addressing gaps through carefully designed studies is essential to ensure effective and practical treatment regimens that can accommodate diverse patient populations.

## Future Directions

The global resurgence of syphilis poses significant public health challenges [5]. Despite penicillin’s efficacy, its limitations—including supply shortages, adherence challenges, and reliance on injectable formulations—underscore the need for alternative antibiotics and improved diagnostics. Addressing these gaps requires a multi-pronged approach that includes innovative diagnostics, population-specific research, and equitable access to antibiotics. Priorities for future research include:

- **Development of Modern Diagnostics:** Integrating direct detection methods, such as PCR-based assays or antigen-detection technologies, will complement traditional serology and improve treatment monitoring.
- **Clinical Trials for Alternative Antibiotics:** Trials are needed to evaluate the safety, efficacy, and dosing of alternatives—such as ceftriaxone, doxycycline, dalbavancin, and cefixime—especially for vulnerable populations like pregnant individuals, neonates, and those with neurosyphilis.
- **Simplified Treatment Regimens:** Shortened or simplified regimens for late latent syphilis will improve adherence and accessibility, especially in resource-limited settings.
- **Pharmacokinetic Studies:** Studies to refine antibiotic dosing for pregnant individuals and neonates will provide evidence for subsequent trials and ensure safe, effective treatments.

Efforts to improve global access to antibiotics must include affordable formulations, strengthened supply chains, and international collaboration. These measures are critical in resource-limited settings, where syphilis remains disproportionately burdensome.

Standardized guidelines and locally adaptable protocols are essential for adopting new therapies and diagnostics. Training programs should emphasize accurate diagnostics, timely treatment, and emerging tools. Incorporating molecular diagnostics into routine care will further enhance precision in treatment evaluation.

Closing these gaps will reduce the global burden of congenital and late-stage syphilis. Sustained investment in research, public health infrastructure, and international collaboration is vital to delivering equitable, effective syphilis care.

## Conclusion

Syphilis remains a pressing global health challenge, with its resurgence exacerbating the burden of congenital syphilis and underscoring the need for modernized approaches to treatment and diagnosis [6, 7]. While penicillin remains effective, reliance on a single therapy has exposed critical gaps in care, particularly for vulnerable populations. Recent advances in antibiotic development and molecular diagnostics offer a pathway to improved outcomes, but their integration into practice requires sustained collaboration among researchers, policymakers, and healthcare providers.

By addressing barriers to access, refining treatment regimens, and expanding diagnostic capabilities, we have the opportunity to significantly reduce syphilis-related morbidity and mortality. Achieving this will not only improve individual outcomes but also contribute to global efforts to curb the resurgence of this preventable disease.

## Key References

- McDonald R, O'Callaghan K, Torrone E, Barbee L, Grey J, Jackson D, et al. Vital Signs: Missed Opportunities for Preventing Congenital Syphilis—United States, 2022. MMWR Morb Mortal Wkly Rep. 2023;72(46):1269–74.
- Tao YT, Gao TY, Li HY, Ma YT, Li HJ, Xian-Yu CY, et al. Global, regional, and national trends of syphilis from 1990 to 2019: the 2019 global burden of disease study. BMC Public Health. 2023;23(1):754.
- Harris E. "Alarming Trend" Persists as Syphilis Cases Swelled in the US in 2022. JAMA. 2024;331(9):725.
- Stafford IA, Workowski KA, Bachmann LH. Syphilis Complicating Pregnancy and Congenital Syphilis. N Engl J Med. 2024;390(3):242–53.
- Clement ME, Okeke NL, Hicks CB. Treatment of syphilis: a systematic review. JAMA. 2014;312(18):1905–17.
- Callado GY, Gutfreund MC, Pardo I, Hsieh MK, Lin V, Sampson MM, et al. Syphilis Treatment: Systematic Review and Meta-Analysis Investigating Nonpenicillin Therapeutic Strategies. Open Forum Infect Dis. 2024;11(4):ofae142.
- Callado GY, Pardo I, Gutfreund MC, Deliberato RO, Holubar M, Salinas JL, et al. Insights Into Treatment Alternatives for Neurosyphilis: Systematic Literature Review and Meta-Analysis. Sex Transm Dis. 2024;51(10):641–7.

## Supplementary Information

Below is the link to the electronic supplementary material.

Supplementary file1 (DOCX 46 KB)

## Data Availability

No datasets were generated or analysed during the current study.
