# Supplementary material for: Expanding Horizons in Syphilis Treatment: Challenges, Advances, and Opportunities for Alternative Antibiotics
Source: Curr HIV/AIDS Rep. 2025 Mar 13;22(1):22. doi: 10.1007/s11904-025-00725-4 (PMC11903563; doi:10.1007/s11904-025-00725-4)
Supplement: Supplementary file 1 — Supplementary file1 (DOCX 46 KB) [file 11904_2025_725_MOESM1_ESM.docx]

### Supplemental Table.

**Supplemental Table 1.** Recent Systematic Reviews with Meta-analyses and Clinical Trials (2019 – 2024)

| Category | Study | Therapies Compared | Population | Outcome |
| --- | --- | --- | --- | --- |
| Systematic Reviews | Callado et al. (2024) (30) | Penicillin vs. ceftriaxone, azithromycin, doxycycline, drug combinations | 27 studies, 6710 participants with non-neurologic syphilis (10 RCTs, 16 retrospective cohorts, 1 prospective cohort) | No superiority of penicillin over ceftriaxone, azithromycin, or doxycycline. |
|  | Callado et al. (2024, neurosyphilis) (31) | Penicillin vs. ceftriaxone, doxycycline | 6 studies for review (2 RCTs, 4 retrospective cohorts); 3 studies for meta-analysis, 236 participants with neurosyphilis (1 RCT, 2 retrospective cohorts) | Similar outcomes between ceftriaxone and penicillin for neurosyphilis. |
|  | Li and Jiang (2020) (45) | Penicillin vs. azithromycin | 7 RCTs, 639 participants | No significant difference in treatment response between penicillin and azithromycin. |
|  | Liu et al. (2022) (46) | Penicillin vs. ceftriaxone, tetracycline, doxycycline, minocycline, erythromycin | 17 studies (3 RCTs, 14 retrospective cohorts) | No significant differences between treatments for syphilis. |
| Recent and Clinical Ongoing Trials | Chen et al. (2023) (92) | benzathine penicillin G vs. benzathine penicillin G + doxycycline x 7 days | Persons with HIV with Early syphilis | Improved 12-month serological response. |
|  | Naokatsu Ando et al. (2023) (58) | High-dose amoxicillin with probenecid vs. low-dose amoxicillin | Non-neurological syphilis | Low-dose amoxicillin not non-inferior to high-dose amoxicillin with probenecid. |
|  | Ubals et al. Trep-AB Study (2024) (84) | Linezolid 600 mg PO BID x **5 days** vs. BPG | Early syphilis | Linezolid inferior to BPG (70% vs. 100% response). Trial stopped for futility. |
|  | Klausner et al. (2024) (78) | Linezolid 600 mg PO BID x **10 days** vs. benzathine penicillin G 2.4 mil IU IM once | Early syphilis | Pilot trial ongoing. |
|  | Taylor et al. (2020) CeBra Trial (80) | Cefixime 400 mg PO BID x 10 days vs. benzathine penicillin G 2.4 mil IU IM once | Early syphilis, Brazil | Phase II trial ongoing. |
|  | Klausner et al. (2021) (79) | Cefixime 400 mg PO BID x 10 days vs. benzathine penicillin G 2.4 mil IU IM once | Early syphilis, 9 sites in US & Peru | Pilot data favorable, now in Phase 3. |
|  | Chen et al. (2023) (92) | Ceftriaxone 1g IM once + doxycycline x 7 days vs. benzathine penicillin G + doxycycline x 7 days | Early syphilis + STI co-infections, Taiwan | Aims to treat syphilis + STI co-infections simultaneously. |
|  | Hla et al. (2024) SCIP trial (93) | Subcutaneous infusion of 7.2 mil IU of benzathine penicillin G once | Non-neurological late latent syphilis, Australia | Phase 2 trial ongoing. |
|  | Pei et al. (2021) | Cefixime 100 mg PO BID x 15 days vs. benzathine penicillin G 2.4 mil IU IM once weekly x 2 weeks | Early symptomatic syphilis | No significant difference in time to rash disappearance, no significant different in time to TRUST reversion. |
|  | Wu et al. (2021) (94) | Minocycline 100 mg PO BID x 28 days vs. benzathine penicillin G 2.4 mil IU IM once weekly x 1-2 weeks | Early syphilis | No significant difference in serological cure rate between penicillin and minocycline. |
|  | Hook et al. (2023) (88) | Benzathine penicillin G 2.4 mil IU IM once vs. benzathine penicillin G 2.4 mil IU IM once weekly x 3 weeks | Early Syphilis | No significant differences between treatments for syphilis. |
